# WHO IDENTIFIES WITH AGE-BASED DISCRIMINATION?

**DOI:** 10.1093/geroni/igae098.1138

**Published:** 2024-12-31

**Authors:** Douglas Hanes

**Affiliations:** Stony Brook University School of Medicine, Stony Brook, New York, United States

## Abstract

**Background:**

Ageism remains a persistent problem that contributes to worse later-life outcomes. Since the COVID-19 pandemic, ageism has received greater attention from activists, researchers, and policymakers. Yet little is known about who identifies with ageism as the basis of their discriminatory experiences, and how people with intersecting marginalized identities endorse ageism as a basis for their discrimination.

**Method:**

Health and Retirement Study (2008–2018, N = 16,250) participants were asked about their experiences of discrimination and the reasons for them, including age and other factors. We analyze which experiences and identities predict endorsement of age as the basis of participants’ discrimination. Multilevel logistic regressions with “discrimination due to age” as the outcome variable were conducted.

**Results:**

Age predicted a small increased likelihood of ageism (OR = 1.08; p < 0.001), but more frequent discrimination decreased it (OR = 0.877, p < 0.001). People of color were less likely to report ageism than non-Hispanic whites (OR = 0.552, p < 0.002), regardless of reporting racist discrimination; whites who reported race-based discrimination had greater odds of also reporting ageism (OR = 1.563, p < 0.001). Women were more likely to report ageism (OR = 1.139; p = 0.002); reporting gender-based discrimination increased ageism probability for both women (OR = 2.318, p < 0.001) and men (OR = 3.037, p < 0.001).

**Discussion:**

Members of certain identity groups and with certain experiences are more likely to understand themselves as subject to ageism than others, which may affect the effectiveness of anti-ageist messaging and interventions.

